# Permeable Asphalt Pavements (PAP): Benefits, Clogging Factors and Methods for Evaluation and Maintenance—A Review

**DOI:** 10.3390/ma17246063

**Published:** 2024-12-11

**Authors:** Maria Sousa, Marisa Dinis Almeida, Cristina Fael, Isabel Bentes

**Affiliations:** 1 Centre of Materials and Civil Engineering for Sustainability (C–MADE), University of Beira Interior (UBI), 6201-001 Covilhã, Portugal; msfda@ubi.pt (M.D.A.); cmsf@ubi.pt (C.F.); 2 Centre of Materials and Civil Engineering for Sustainability (C–MADE), University of Trás-os-Montes and Alto Douro (UTAD), 5000-801 Vila Real, Portugal; ibentes@utad.pt

**Keywords:** permeable asphalt pavement, clogging, X-ray computed tomography, maintenance

## Abstract

Permeable asphalt pavement (PAP) is an efficient solution to stormwater management, allowing water to infiltrate through its layers. This reduces surface runoff and mitigates urban flooding risks. In addition to these hydrological benefits, PAP enhances water quality by filtering pollutants such as organic and inorganic materials and microplastics. However, clogging from sediment accumulation in the pavement’s void structure often impairs its performance, reducing infiltration capacity. This review addresses several issues related to PAP, including the factors that contribute to pavement clogging and evaluates current and emerging maintenance strategies, including manual removal, pressure washing, regenerative air sweeping and vacuum truck utilization. Additionally, different methods of assessing clogging using innovative technology such as X-Ray Computed Tomography (CT), as well as a summary of the software used to process these images, are presented and discussed as tools for identifying clogging patterns, analyzing void structure and simulating permeability. This review identifies gaps in existing methodologies and suggests innovative approaches, including the creation of self-cleaning materials designed to prevent sediment buildup, biomimetic designs modeled after natural filtration systems and maintenance protocols designed for targeted clogging depths, to support the optimization of PAP systems and promote their adoption in resilient urban infrastructure designs in alignment with Sustainable Development Goals (SDGs).

## 1. Introduction

The intense urban growth associated with the displacement of populations to cities has led to a progressive increase in soil sealing. The massive development of urban areas is one of the main causes of the profound alteration of the hydrological cycle, which, together with climate change, increases the risk of flooding, as high-intensity, short-duration rainfall events occur more frequently. These phenomena have a considerable impact on the management of water resources and urban supply and drainage systems, as well as causing financial losses and threats to public health and safety [1,2].

The use of Sustainable Urban Drainage Systems (SUDSs) is intended to mitigate the issues resulting from flooding by managing stormwater in cities. Permeable pavements are included in SUDSs and are one of the solutions that have been applied around the world, having a structure which, besides withstanding traffic loads, simultaneously allows stormwater to infiltrate for various purposes, including temporary storage, dispersal into the soil or collection for treatment and reuse [3,4].

Permeable pavements are directly exposed to the environment and are subject to human activities. The deposition of sediments, organic material and particles found in stormwater runoff in the pavement layers leads to clogging and, consequently, obstruction of the voids in the asphalt mix layers. This causes a reduction in its infiltration capacity, reducing its hydraulic functionality [5].

Therefore, during the period of its use, maintenance and monitoring is required to ensure that all layers retain their drainage effectiveness during their useful life [6].

A laboratory study investigated mixtures composed of stamp sand and acrylonitrile styrene acrylate (ASA) plastic waste as alternatives to conventional asphalt, highlighting their high permeability, thermal resistance and potential to reduce environmental impacts in pavements [7].

The study highlights that ASA mixtures have a water permeability coefficient 6 to 10 times higher than that of conventional asphalt mixtures. This high permeability makes these mixtures less susceptible to clogging in porous pavements, facilitating water drainage and improving their performance in drainage areas [7].

The purpose of this literature review is to raise relevant questions about the clogging process in permeable pavements, identifying its characteristics, the factors that influence it and the different types of clogging solutions. In the first part of this review, the definition of permeable pavements is presented. The second part identifies some factors that influence clogging. In the third part, prevention and mitigation techniques of clogging are exposed. Finally, conclusions of the review research are presented.

## 2. Methodology

In this literature review, some inclusion criteria for literature review research were defined; for example, the language chosen was English because it is a universal language in the scientific environment, the databases chosen were MDPI [8] and ScienceDirect [9] because they contain numerous scientific journals and magazines, and some documents were selected (review articles, research articles and book chapters in the theme areas of Environmental Science, Materials Science and Engineering).

**Figure 1:**
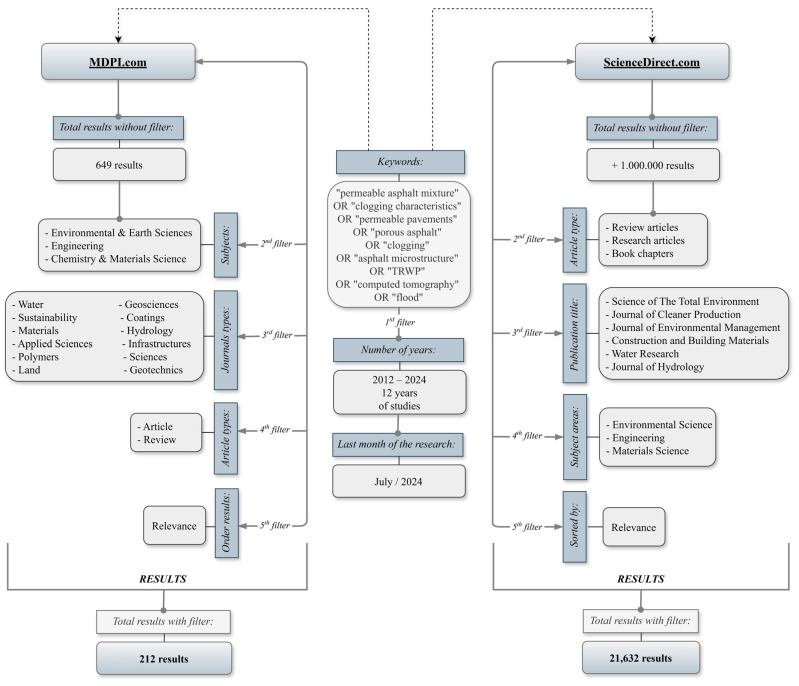
Study structure diagrams developed for MDPI and ScienceDirect.

**Figure 2:**
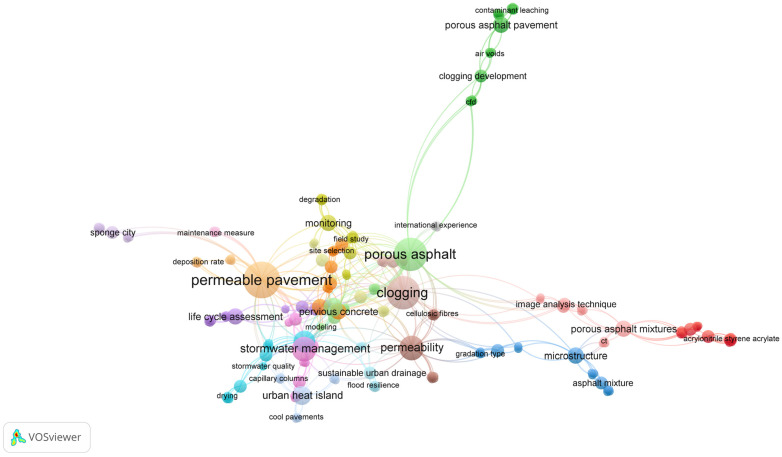
Keywords connections diagram. Created in VOSviewer.com (https://www.vosviewer.com, accessed on 17 November 2024) [10].

The data selection and collection processes were carried out in July 2024, and in the first step of the process the keywords used in the abovementioned databases were identified, such as “permeable asphalt mixture” OR “clogging characteristics” OR “permeable pavements” OR “porous asphalt” OR “clogging” OR “asphalt microstructure” OR “TRWP” OR “computed tomography” OR “flood”. In the second stage, the filters available in the databases were applied; for example, the periods of the research years, the types of papers, the types of publication and the subject areas, among others, as shown in Figure 1. The structural diagrams of the study developed in MDPI and ScienceDirect are presented. Figure 2 illustrates a diagram highlighting the connections between different keywords.

The search for papers was carried out over a study interval of 12 years, using as a study base a permeable asphalt pavement material that can become completely clogged after a period of 12 years [11].

The selected papers contain information on permeable pavements, focusing on several materials like porous concrete or porous asphalt. This study will focus on porous asphalt. Therefore, general contents are considered, such as clogging, analysis methods and types of maintenance solutions. Papers that include other approaches were excluded from this study.

## 3. Permeable Asphalt Pavements (PAPs)

A first analysis of the papers reveals the significance of permeable pavements in urban applications. This section aims to provide a comprehensive understanding of permeable pavements by addressing several critical aspects. This part discusses their definition, benefits and the challenges posed by clogging. It also reviews methods for evaluating clogging, including advanced imaging techniques, and outlines maintenance strategies to ensure their long-term functionality and effectiveness in sustainable urban development.

### 3.1. Definition

Permeable or porous pavement exemplifies a concept known as Sustainable Urban Drainage Systems (SUDSs). The technique was originally created in the late 1980s in the United Kingdom and The SUDS Manual can be found on the Construction Industry Research and Information Association (CIRIA) website [12]. The aim of water quality control through SUDS techniques is to avoid the concentration of large volumes of water in each area to improve the biodiversity and the environment for living beings [13,14,15].

Permeable asphalt pavements (PAPs) are made up of several layers with a fully porous structure allowing precipitated water to infiltrate into the subsoil, if it has drainage capacity, or store it in a reservoir for future use [16].

The difference between permeable and traditional asphalt mixtures is related to the air void content of these pavement layers. PAP usually has air voids of 15 to 20% in comparison to impermeable pavements that have air voids of 3 to 6% [17,18,19].

In the literature, the most used permeable solutions in pavements surface layers can be diverse, comprising porous concrete (PC), porous asphalt (PA), permeable interlocking concrete pavers (PICPs) or permeable unit pavers (PUPs) and concrete and plastic grid pavers (CGPs and PGPs) [20,21].

### 3.2. Benefits

Permeable asphalt pavements (PAPs) enable the infiltration of rainwater, effectively mitigating surface runoff and reducing the incidence of urban flooding due to their high drainage efficiency. In addition, these pavements contribute to minimizing the urban heat island effect and provide evaporative cooling, thereby enhancing the resilience of communities to climate change. They also function as pollutant filters, improving water quality and supporting ecosystem health.

These pavements provide in situ restoration of the hydrological cycle in urban areas [21]. At the same time, infiltrated water allows aquifers to recharge and make more water available to nearby trees and vegetation [22,23], contributing to the management of atmospheric humidity levels and reducing the urban heat island effect due to its structure with a high void ratio, which reduces the energy stored in the pavement and allows for rapid cooling through evaporation and the availability of moisture near the surface layer [24,25,26,27,28].

It should be noted that in a conventional bituminous pavement, surface runoff can be greater than 90% of the precipitation [29,30].

The higher percentage of infiltration recorded with PAP leads to a reduction in surface runoff and, consequently, a reduction in the risk of flooding in urban areas, the spray effect and aquaplaning, increasing the safety of road users [13,16].

Noise reduction is also one of the benefits highlighted by permeable pavements, due to their high percentage of voids. The voids in the surface layer absorb a large amount of noise generated by the interaction between the vehicle’s tires and the pavement [31,32,33]. In addition, as a result of high porosity and its macrotexture, PAP provides good skid resistance [13].

Permeable pavements are also a solution for controlling tire wear pollutants, fuel residues, vehicle oil and gas leaks, materials from road pavement surface courses, and winter sand and salt applications carried by surface runoff on pavements. Therefore, permeable pavements can act as a means of managing these pollutants that accumulate in the pavement layers over time [16,34,35]. The friction between the tire and the asphalt surface leads to dust and road material formation, known as Tire and Road Wear Particles (TRWPs) [36,37,38]. These particles are classified as Microplastic Particles (MPs), and their dimensions can range from 5 mm to 1 µm [34].

Thus, PAP is defined as a primary means of managing urban stormwater [39,40]. However, as pollutants accumulate in the layers, there is clogging, the pavement’s permeability decreases and, consequently, its infiltration capacity and lifespan is reduced [40].

### 3.3. Clogging

Clogging is the accumulation of sediment particles in surface runoff that deposit and obstruct the voids in the permeable pavement [41]. The primary sources of these sediments are particles from tire wear or asphalt material [11,42], fuel leaks [42], winter maintenance (e.g., salt and sand application), the presence of trees and land use around the pavement. Additionally, clogging is also caused by the formation of biofilms on the surfaces of the void walls [11].

Sediment particles the same size as the pavement voids will cause more blockage as they are absorbed into the surface layer. In contrast, smaller and finer particles will move into the deeper pavement layers [11]. 

Tire and Road Wear Particles (TRWPs) are created by friction between tires and asphalt, road marking paints and vehicle parts [34,43,44]. TRWPs are suspected to be a significant environmental pollutant due to their high abundance in air, water, and sediments [44,45]. 

Although TRWP emissions are very high in Europe, approximately 1.33 × 10^5^ tons/year [45], other studies estimate that the per capita production of TRWPs is between 0.2 and 5.5 kg/year [46].

It has also been observed that microplastic particles, with dimensions up to 10 μm, are released from asphalt on roads and car parks [34].

Several studies concluded that the tire debris material retained by permeable pavements constitutes a non-negligible proportion of the total mass of all pollutants and thus have found that permeable pavements can retain up to 93% of the total load of suspended pollutants in water [47]. It is, therefore, understood that permeable pavements are systems that can manage pollutants present in stormwater as well as flood flows [48].

This way, studies allowed the approximate estimation that the retention of MPs was significant in terms of the amount of MPs that could be generated in roads. It is estimated that other types of plastics were also found in the samples, but at concentrations 49 times lower than that of tire debris particles. Therefore, permeable pavements can serve as a management system for MPs and other pollutants [34].

However, for better understanding, when analyzing the parameters and characteristics of clogging in permeable asphalt pavements, it is essential to clarify the causes of the clogging process of the permeable asphalt mixture through evaluation methods such as permeability tests, pavement microstructure analysis and others [40].

### 3.4. Methods of Evaluating Clogging

A better understanding of the clogging process in permeable pavements is crucial, particularly its origin, the size and distribution of sediments in the voids, their internal location in the layers and their evolution [40].

The permeability test determines the pavement’s permeability coefficient, that is, the ease with which water infiltrates the pavement. This assessment is generally carried out using constant head or variable head equipment. In the latter, the flow time of a water column is measured [40]. Although the permeability test is an adequate method of assessing the infiltration capacity of pavement surfaces, the decrease in permeability alone does not allow the characterization of the clogging type in the pavement layers [49].

X-Ray Computed Tomography (CT) is one of the most used techniques for microstructural analysis in image processing to check for obstructions in pavement voids [49,50,51,52].

The three-dimensional analysis of an asphalt mixture through CT images is advantageous for determining the correlation between its mesoscopic structure and macroscopic properties. After segmentation of the CT images, it is essential to distinguish the aggregates from the air voids in the asphalt mixture [50].

**Figure 3:**
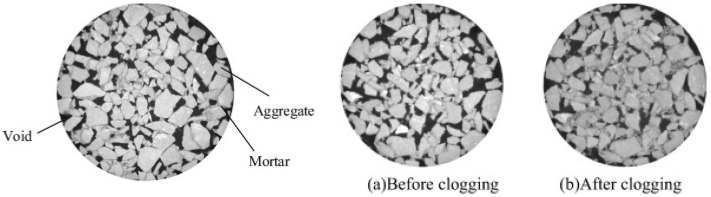
CT image [40].

The result of a CT image is generally a grayscale image, where each gray level represents the X-ray absorption capacity of different materials, which is directly related to their density. Aggregates usually appear lighter gray with a higher density value, while air voids appear darker gray with a lower density value, as presented in Figure 3 [53,54,55].

Once the CT images have been obtained, digital image processing techniques must be applied, typically with the support of software. MATLAB is one solution used to define the characteristics of the air voids in the sample using intensity transformation, grayscale top-hat and bottom-hat transformations, threshold segmentation, morphological smoothing and specimen reconstruction [56].

An alternative image processing software that can process images is ImageJ, a non-destructive method that uses virtual slicing to analyze the properties of air voids in porous asphalt. Using this software, the distribution and properties of air voids, such as number, shape and size, can be analyzed at different angles in the sample [51].

Another example of three-dimensional asphalt mixture reconstruction using X-Ray Computed Tomography (CT) images is a local threshold method based on the Monte Carlo embedded segmentation method. This method constructs a three-dimensional model of the mesoscopic structure to investigate the spatial variation of different components, identifying the multiphase components of the asphalt mixture [50].

**Figure 4:**
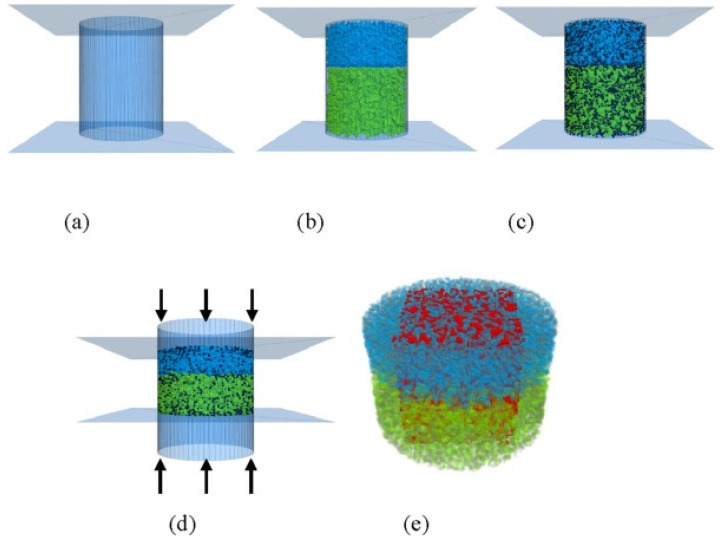
Virtual reconstruction of an double-layer asphalt mixture through the software DEM-CFD implemented in PFC 3D version 4.0: (**a**) creation of the virtual steel model; (**b**) introduction of coarse aggregates, represented by the colors blue and green; (**c**) addition of asphalt mastic, represented by the color black; (**d**) vertical compaction of the asphalt mixture; (**e**) representation of air voids, highlighted in red. [57].

The Discrete Element Method–Computational Fluid Dynamics (DEM-CFD) model implemented in PFC 3D version 4.0, can numerically simulate the clogging of porous asphalt pavements under gravity and water flow. This method analyzes the sediment size, distribution and infiltration velocity and characterizes the voids in the pavement, allowing a detailed analysis of the porous pavement structure. An example of the model is presented in Figure 4 [57].

**Figure 5:**
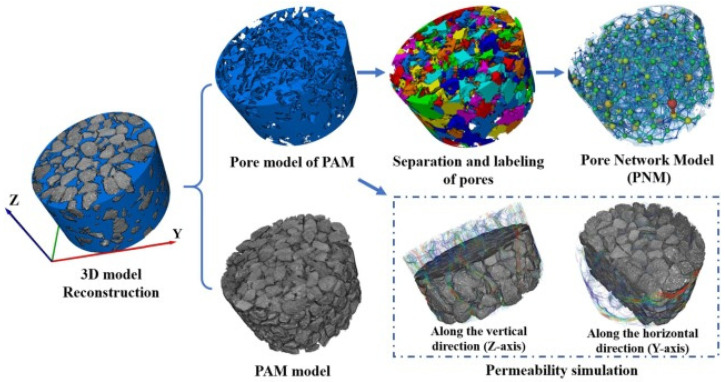
A 3D model pore structure analysis produced through the software Avizo: CT scanning and 3D model reconstruction to analyze the structural characteristics of porous asphalt mixture (PAM) and permeability simulation using the absolute permeability module [58].

The Avizo software can process and analyze computed tomography images, evaluating porosity in 2D and pore structure in 3D. It includes functions to detail parameters such as porosity, type, number, diameter and shape of voids, in addition to allowing permeability simulations with its hydraulic module. The results indicate that interconnected porosity is essential for infiltration capacity, while pore and throat sizes are secondary factors. An example of the model is presented in Figure 5 [58].

**Figure 6:**
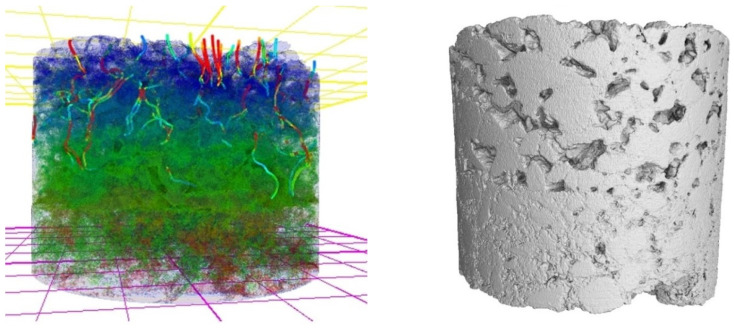
A 3D model produced through the software VGStudio MAX version 2023.4: reconstruction of the 3D model of an asphalt mix, with the aggregates represented by the blue and green colors and simulation of the permeability illustrated by the flow in the vertical direction, represented by colored lines.

The VGStudio MAX software version 2023.4 allows the creation of Surface Tessellation Language (STL) files to represent surface sections and includes functions such as data filtering and application of a three-dimensional median filter [59]. In addition, it assists surface sectioning to determine volumetric data and performs detailed volumetric analyses, including porosity analysis and permeability simulations [60]. Below is an example of a simulation performed by the software VGStudio MAX, presented in Figure 6.

### 3.5. Maintenance Techniques

The service life of a permeable pavement is related to the service period (years) and the reduction in its infiltration capacity due to clogging to a level where it can no longer provide this function in the event of precipitation. The clogging hydraulically restricts water movement through the pavement; hence, maintenance is necessary to restore its permeability [61].

Therefore, the service life of a permeable pavement is expected to be shorter than that of an impermeable pavement. Studies indicate that the estimated service life of a permeable pavement is typically 15 or 20 to 35 years [62]. However, clogging can occur after 5 to 10 years of use [63].

Effective maintenance techniques include ensuring hydraulic functionality and water quality through stormwater control. The recovery of pavement permeability can be achieved by applying different small-scale and full-scale maintenance techniques to remove sediments and debris that clog the air voids [64].

Some of the maintenance measures used include high-pressure water washing (HW), low-pressure suction (LS), high-pressure air flushing (HA), mechanical street sweeping, vacuum and regenerative-air street sweeping, hand-held vacuuming, milling of porous asphalt [64,65], moistening followed by sweeping, sweeping followed by suction, suction alone, high-pressure water jets combined with simultaneous suction [66], manual pressure washer and vacuum, leaf blower and push broom and vacuum-assisted street sweepers [67].

The recommended frequency of maintenance varies from once a year [66] to two or four times a year [42], depending on location and climatic conditions. Frequent maintenance is required in areas with higher pollutant concentrations and deposition rates [62].

## 4. Results Analysis of Clogging in Pavements

The service life of permeable pavement is directly related to several factors, including the location of the application, the durability of the material used, the quality of the materials applied, and proper maintenance. Table 1 summarizes research into the type of pavement, the maintenance techniques used. In addition, the effectiveness evaluation of the maintenance methods used on different types of permeable pavements is highlighted.

Among the studies analyzed, one examined the clogging of various types of permeable pavements in different locations across countries such as Sweden, the United States, China and Spain, where these pavements are implemented in residential streets, parking lots, community centers and other similar areas [64,65,68,69,70,71,72].

Each location exhibits specific sedimentation characteristics, including the presence of organic sediments, fine sediments [64], construction debris [71] and natural sediments [65], as well as clogging factors associated with freeze–thaw cycles during the winter [70].

The primary reason for the poor performance of permeable pavements is the lack of regular maintenance, resulting in prolonged sediment accumulation in the voids, which compromises their functionality and shortens their lifespan. Research shows that after 10 years, these pavements may reach the end of their service life due to sediment buildup [71].

However, after the implementation of regular maintenance on permeable pavements, such as the use of regenerative air sweepers, high efficiency was observed, achieving infiltration levels of up to 100% [64]. Regenerative air sweepers are among the best options, but other maintenance methods can also be effective, such as the use of high-pressure water jets and mechanical brushing.

## 5. Discussion and Conclusions

This paper reviews several studies based on the appliance of permeable pavements in urban areas, with a focus on their hydrological benefits and maintenance challenges of permeable asphalt pavements (PAPs). Permeable pavements represent a critical component in sustainable urban development, mitigating issues in stormwater management, groundwater recharge and pollutant filtration. Although they offer advantages, clogging remains a significant challenge, requiring advanced microstructural analysis and maintenance techniques.

Various software programs were also mentioned, which, from the images collected by X-Ray Computed Tomography (CT), have the function of detailing parameters such as porosity, type, number, diameter and shape of voids, in addition to allowing permeability simulations.

Scientific advancements, including CT and advanced image processing software, provide valuable tools for understanding and addressing clogging mechanisms. However, the development of innovative solutions, such as self-cleaning materials to repel or prevent sediment accumulation and biomimetic designs inspired by nature that replicate natural filtration systems, is essential for minimizing maintenance requirements and extending pavement lifespan.

The case study analysis emphasized the importance of periodic maintenance to preserve the durability and lifespan of permeable pavements by addressing void blockages, maintaining infiltration efficiency and minimizing deep obstructions. Advanced methods are required for deeper blockages, with maintenance solutions tailored to the pavement type, obstruction cause and depth. Common methods include manual removal, suction and pressure washing, regenerative air sweeping and regular surface cleaning. In some cases, combining methods is essential for optimal maintenance efficiency.

Further investigations should address developing cost-effective, environmentally sustainable maintenance methods and integrating permeable pavements into broader urban water management systems. Incorporating these innovations, permeable pavements can further their contribution to resilient, climate-adaptive cities, aligning with Sustainable Development Goals (SDGs).

## Figures and Tables

**Table 1:** Summary of pavement types and maintenance effectiveness.

Type of Pavement	Maintenance Method	Efficiency	References
Concrete grid pavers (CGPs) (Residential street)	Manual removal of upper 2 cm	Infiltration improvement was limited (9–55%) and ineffective for deep clogging; manual removal failed.	[64]
Permeable interlocking concrete pavers (PICPs) (Residential street)	Manual removal of upper 2 cm	Manual removal was effective for surface cleaning (61–100% infiltration) but ineffective for deep clogging (4–22% infiltration).	[64]
Porous asphalt (Residential street)	Vacuuming, pressure washing, milling	Milling restored infiltration, while pressure washing was partially ineffective (3–5% improvement).	[64]
Permeable interlocking concrete pavers (PICPs) (Parking lot)	Regenerative air street sweeper or mechanical street sweeper	The regenerative air sweeper was highly efficient (16–100% infiltration), surpassing the mechanical sweeper (2–68%).	[64]
Permeable interlocking concrete pavers (PICPs) (Community center parking lot)	Vacuum truck with 1 pass and 3 passes	Percentage of infiltration after maintenance: Vacuum 1 pass: 100%. Vacuum 3 passes: 76–100%.	[64]
Concrete pavement (PC), Interlocking impermeable concrete brick pavement (IIC), Interlocking permeable concrete brick pavement (IPC)	High-pressure water washing (HW), Low-pressure suction (LS), High-pressure air flushing (HA)	Maintenance solutions varied by pavement type, with LS, HW and HA proving most efficient.	[65]
Porous pavement of grass pavers (Parking lot) Traditional asphalt pavement (Parking lot)	Regular sediment removal and cleaning of pavement voids, along with surface waste maintenance, are essential	Porous pavements reduce runoff (up to 93%) but require regular maintenance, unlike asphalt, which increases runoff and turbidity.	[68]
Porous asphalt (PA) PA with 20% porosity and PA with 25% porosity	Surface cleaning Surface cleaning with water pressure Deep vacuuming	Simulation results: Unclogged PA 20%: Porosity of 19.02%. Unclogged PA 25%: Porosity of 25.68%. Clogged PA 20%: Porosity reduced to 16.88%. Clogged PA 25%: Porosity reduced to 23.72%.	[69]
Porous asphalt mixtures (PAMs) Performance evaluation of PAMs in cold regions	CT scan and surface texture analyses, binder extraction and recovery, along with regular snow and ice removal maintenance	Reduced surface texture quality, increased binder stiffness and brittleness, with altered air void distribution.	[70]
Hydrological performance (Car park “Las Llamas”) Interlocking Concrete Block Pavements (ICBPs) Permeable Pavement Systems (PPSs)	Permeability tests using permeameter, Spanish standards and ASTM test.	After 10 years without maintenance, the clogged pavement reached the end of its operational life.	[71]
Simulation study: Permeable Pavements (PPs)	Two rainfall simulators were built to study sediment gradation effects on clogging (University—UDC) and pollutant reduction at different clogging levels (University—UPV).	Surface vacuuming reduced permeability by 22–99% due to sediment load, with recovery ranging from 8–100% post-cleaning.	[72]
